# Evaluation of a Gamification-Based Intervention to Enhance Intergenerational Communication: Pre-Post Mixed Methods Evaluation

**DOI:** 10.2196/77190

**Published:** 2026-07-31

**Authors:** Eliza Lai-Yi Wong, Clement Cheuk Wai Ng, Dorothy Yingxuan Wang, Carol Ka Po Wong, Shirley Shuk Kuen Lui, Annie Wai-Ling Cheung, Eng-kiong Yeoh

**Affiliations:** 1 Centre for Health Systems and Policy Research, The Jockey Club School of Public Health and Primary Care, Faculty of Medicine, The Chinese University of Hong Kong, Room 418, School of Public Health Building, Prince of Wales Hospital, Ngan Shing St, Sha Tin, China (Hong Kong), 852 22528772; 2 The Jockey Club School of Public Health and Primary Care, Faculty of Medicine, The Chinese University of Hong Kong, Hong Kong, China (Hong Kong)

**Keywords:** intergenerational communication, health, gamification intervention, social isolation, social connection, aging

## Abstract

**Background:**

Intergenerational communication has been recognized as a key factor in enhancing social connections among older adults by improving the quality of interactions, which is a crucial component of healthy aging. However, effective interventions to foster intergenerational communication and engagement remain scarce at this stage.

**Objective:**

This study examined the effects of a gamification intervention for intergenerational dialogue on intergenerational communication and health-related capacity across 3 age cohorts and further analyzed the differential impacts of intervention characteristics on the game experience, in terms of satisfaction and engagement.

**Methods:**

A 2-stage exploratory method was adopted with a cocreation approach to develop a theoretical framework of intergenerational dialogue for the gamification-based intervention, the Healthy Aging in Dialogue Tabletop Game, followed by a mixed methods study involving pre- and postintervention surveys and qualitative interviews to evaluate the effectiveness of the intervention in the young (aged 18‐39 years), middle-aged (aged 40‐64 years), and older adult (aged ≥65 years) groups. Intergenerational communication was assessed using tools measuring satisfaction and competence, whereas health-related capacity was evaluated using health literacy and patient enablement tools.

**Results:**

A total of 68 respondents completed the intervention and evaluation, with 23, 14, and 31 respondents in the young, middle-aged, and older adult groups, respectively. This intergenerational intervention yielded notable benefits in intergenerational communication satisfaction (Cohen *d*=0.745), communication competence (Cohen *d*=0.377), and health literacy (Cohen *d*=0.409), especially among older adults. Patient enablement was significantly higher in older adults (mean score 6.10, SD 2.18) than in young adults (mean score 4.22) (*P*=.04). In addition, this study highlighted the importance of leveraging gamification to enhance intergenerational communication and engagement, ultimately combating social isolation among older adults.

**Conclusions:**

The intergenerational dialogue tabletop game provided an enjoyable platform for cross-generational dialogue and enhanced each age group’s communication competence, allowing participants to contribute and learn from one another. The findings provide additional evidence for aging research and shed light on approaches to foster social interaction and connection, thereby promoting health and active aging.

## Introduction

Rapid technological advancement, urbanization, and aging have significantly reshaped social dynamics, family structures, and community engagement, leading to fewer face-to-face interactions. Evidence shows that digital communication increasingly replaces face-to-face interactions [1], with individuals often withdrawing from in-person contact due to technology use and fast-paced lifestyles [2]. These societal shifts, combined with changing family structures linked to population aging, contribute to reduced intergenerational engagement and weakened community ties [3]. Among these transformations, social isolation has emerged as a critical concern since the 1990 s due to its profound implications across levels of society, including individuals, families, workplaces, disease groups, and broader society [4,5]. Social isolation is defined as the objective absence or insufficiency of meaningful and sustained communication [6], reflected in having too few interactions with others. It has become a priority global health issue recognized by the World Health Organization (WHO) and the United Nations, with an estimated 25% of older people and 15% of adolescents experiencing social isolation [7] because it has been linked to numerous adverse outcomes documented by a growing body of evidence, such as mental health decline, depression, dementia, cardiovascular disease, stroke, falls, reduced quality of life, hindered active aging, and increased mortality rates [8,9]. Our preliminary work shows that prevailing circumstances, such as pandemics, climate change, policies, family crises, and health deterioration, further intensify social isolation [10,11]. Loneliness, by contrast, represents a distressing feeling arising from a perceived and experienced lack of interaction [6]. Although social isolation is an objective form of social disconnection, whereas loneliness is a subjective form, they are strongly related [12], and both forms of disconnection carry significant health risks [7,13].

Many intergenerational programs have been shown to mitigate social isolation and loneliness by strengthening cross-age communication and expanding older adults’ social networks [14-16]. Intergenerational communication, defined as the exchange of ideas, values, and experiences between different age groups [17], has been recognized as a key factor in addressing social isolation by fostering mutual understanding and improving the quality of intergenerational interactions within an intergenerational program [15]. Existing intergenerational programs mainly focus on structured connections and individual-based interactions (eg, one-way storytelling, companionship for social and health support), and community-based activities (eg, group-based activities related to arts, gardening, physical activity, cooking, digital games, volunteering, and neighborhood support) [14-16]. The conversation-based program remains limited, despite being the central component of social connection. Without engaging in dialogue topics or accommodating communication practices, social isolation can be perpetuated. Key components of communication include listening, observing verbal and nonverbal cues, and expressing thoughts and emotions through both verbal and nonverbal channels [18], all of which are essential for achieving mutual understanding, building a trusting relationship, and fostering an empathetic and caring environment [19]. When individuals lack adequate communication skills or have limited exposure to diverse conversational partners, they may struggle to accurately interpret others’ cues or express themselves effectively. This is particularly evident in intergenerational contexts. Giles and Gasiorek [20] found that younger adults often overaccommodate older individuals through overprotection, whereas older adults tend to underaccommodate younger individuals by voicing harsh criticisms of their generation. A lack of intergenerational understanding may deepen generational divides, leading to age stereotypes that may contribute to nonaccommodation [21]. Without engaging in dialogue topics or practicing accommodating communication strategies, social isolation can be reinforced. Furthermore, most programs are time-limited, and older adults often lose access to the platforms and opportunities that enable ongoing interaction with younger people, leading to a decline in sustained engagement.

Given that the intervention was explicitly designed to structure and enhance intergenerational dialogue, this study focuses on proximal communication outcomes, specifically communication competence and communication satisfaction, as direct indicators of interaction quality. Although social isolation and loneliness motivate this work as broader public health concerns, these outcomes are conceptualized as distal and likely to emerge through sustained improvements in the communication process over time. Accordingly, this study evaluates the effects of the intervention on communication-related outcomes, providing a necessary foundation for future research to examine downstream impacts on social isolation and loneliness. This paper addresses this gap by developing a structured intergenerational tabletop game as a repeatable, low-cost platform that provides guided conversation topics and supports the four key communication components as intervention mechanisms that facilitate meaningful interaction beyond formal programs: guided conversational exchange; shared problem-solving through cooperative tasks; perspective-taking and mutual learning across generations; and opportunities to practice and build confidence in communication competence. Additionally, the intervention embedded health content to enhance health literacy and enablement, supporting participants’ capacity to sustain and apply communication gains, as poor health literacy has been recognized by the WHO as a key driver of social isolation and a diminished capacity for social participation [3].

## Methods

### Overview

A 2-stage exploratory method was used to develop intergenerational dialogue prototypes for the gamification-based intervention and to evaluate its effectiveness on the communication process. Gamification was adopted to incorporate game-based intervention elements such as points, leaderboards, and badges into a nongame context to enhance the engagement experience [22]. This study investigated the effectiveness of the “Healthy Aging in Dialogue Card Game” on intergenerational communication, health, and game experience in three age cohorts: 18‐39 years, 40‐64 years, and ≥65 years. The objectives were to evaluate communication skills (communication competence and satisfaction), health literacy, self-efficacy, and engagement in the game (fun, encouragement to communicate, health knowledge building, and satisfaction) across age groups in the pre- and postsurveys.

### Study Population

#### Overview

Participants who were Hong Kong residents and spoke Cantonese as their native language were eligible for the study, while those without the cognitive capacity to provide consent were excluded. Recruitment of young adults aged 18‐39 years and middle-aged adults aged 40‐64 years was conducted via mass mailings at universities and social media, including Facebook (Meta Platforms, Inc) and Instagram (Meta Platforms, Inc). Older adults aged 65 years or older were recruited by nongovernmental organizations via flyers and a multicommunity-center approach.

#### Stage 1: Cocreation and Prototype Development for Intergenerational Dialogue

The development of the intergenerational dialogue intervention followed the revised Medical Research Council guidance for complex interventions [23], with an emphasis on theory-driven design, stakeholder input, and iterative refinement. A 4-member cocreation team, comprising an academic with training in nursing and public health (ELYW), a social worker, a public health practitioner (CKPW), and 1 potential end user (SSKL), led the multistage development process.

#### Theoretical Framework: Listening, Observing, Understanding, and Expression

The intervention was theoretically informed by the Listening, Observing, Understanding, and Expression (LOUE) framework, which integrates core verbal, nonverbal, and emotional communication processes [18]. LOUE was selected because it aligns closely with the goals of intergenerational dialogue, where effective communication depends not only on verbal exchange but also on emotional attunement, reciprocal self-disclosure, and perspective-taking. Specifically, active listening and empathic engagement are fundamental to building understanding and trust in interpersonal communication [24], while emotional attunement enables individuals to perceive and respond sensitively to others’ affective states [25]. Perspective-taking further supports interpersonal connection by allowing individuals to understand others’ thoughts and viewpoints, and reciprocal self-disclosure fosters relational closeness and social bonding [26]. In this study, LOUE served as a design framework to guide the generation, classification, and structuring of dialogue prompts, ensuring that gameplay systematically exercises these 4 communication skills.

#### Identification of Dialogue Categories and Topic Generation

**Figure 1.**
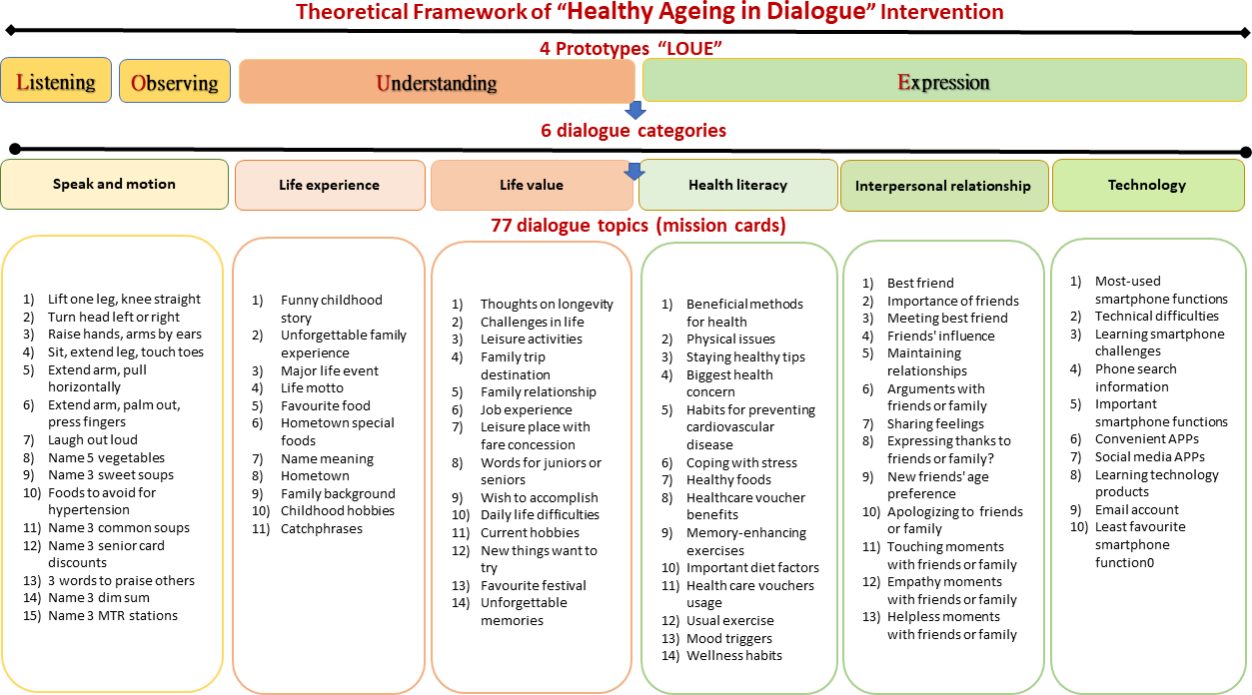
Theoretical framework of the Healthy Aging in Dialogue intervention. MTR: metro.

At the identification stage, the cocreation team reviewed evidence from intergenerational communication and health promotion programs [27,28] and collaboratively identified recurring thematic domains relevant to everyday intergenerational interactions. Through iterative discussion and clustering of draft prompts, six dialogue categories, defined as thematic groupings of communication prompts, were agreed upon: (1) Speak and Motion, (2) Life Experience, (3) Life Values, (4) Health Literacy, (5) Interpersonal Relationship, and (6) Digital Literacy (Figure 1). Building on these categories, the team generated an initial pool of conversation prompts and refined them based on relevance, clarity, and suitability for group dialogue. This process resulted in 77 dialogue topics, which were further refined through a design review to ensure balanced representation across categories and suitability for delivery in a game-based format.

#### Operationalization as Mission Cards and Intervention Structure

The 77 dialogue topics were operationalized as mission cards, each containing a structured prompt designed to elicit turn-taking dialogue and reflection. Mission cards were evenly distributed across the 6 dialogue categories. Each card primarily targeted 1 LOUE dimension (eg, listening or expression), while still engaging all 4 communication skills through interaction and response. This structure ensured that each game turn focused on a specific topic while cumulatively strengthening intergenerational communication competencies.

#### Gamified Tabletop Intervention Design

These mission cards were embedded within a gamified tabletop board game titled the “Healthy Aging in Dialogue Tabletop Game.” The intervention consists of players taking turns drawing mission cards, completing dialogue tasks, and accumulating points through participation rather than correct performance. Each player selects a chess piece at the start of the game to represent equal participation and balanced turn-taking, highlighting intergenerational communication as a reciprocal process.

Gamification elements were incorporated to enhance engagement, self-expression, and motivation [29]. A point-based system (1‐3 points, reflecting increasing depth of conversational engagement, ranging from factual exchanges to meaning-making and reflective discussion) was used. Evidence suggests that such gamification strategies can promote interaction, maintain autonomy, and sustain engagement [29,30]. Importantly, 58 of 77 (75%) mission cards require the active involvement of multiple players, such as inviting adjacent or all players to respond, thereby encouraging listening, observing others’ perspectives, and shared reflection [31-33].

#### Game Format and Play Procedure

**Figure 2.**
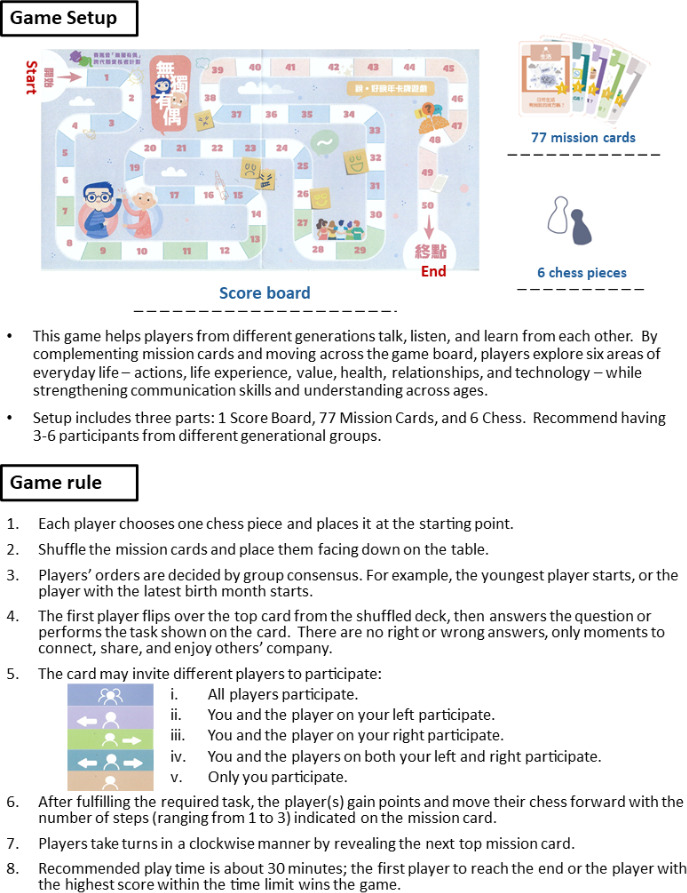
Healthy Aging in Dialogue Tabletop Game.

The intervention is designed for 3‐6 players aged 11 years or above, with each group comprising at least 1 young adult (18‐39 years), 1 middle-aged adult (40‐64 years), and 1 older adult (≥65 years). A typical game session lasts 20‐30 minutes, or until a preset endpoint is reached, to maintain attention and prevent conversational dominance. Players take turns clockwise, draw mission cards, and complete tasks within approximately 1‐2 minutes per turn. There are no correct answers, and judgmental feedback is discouraged to maintain a respectful and inclusive dialogue environment. Details of the game setup and rules are provided in Figure 2.

#### Stage 2: Mixed Methods Evaluation of the Effectiveness of the Gamification-Based Intervention

A mixed methods study method consisting of a 2-phase longitudinal survey and follow-up qualitative sessions involving focus group or individual interviews was conducted on-site to evaluate the effectiveness of the gamification-based intervention—the Healthy Aging in Dialogue Tabletop Game. Before the intervention, all participants completed the baseline questionnaires independently or with assistance from a trained research assistant. Immediately after the intervention, they completed the follow-up questionnaire. A minimum sample size of 63 was recommended to detect a medium effect size of 0.5 at a significance level of .05 and statistical power of 80%, with the assumed SD of 1 [34]. The questionnaire consisted of the following four sections: (1) intergenerational communication, (2) health-related capacity, (3) game experience (postintervention only), and (4) demographics (baseline only).

### Primary Outcome Measure

Intergenerational communication was assessed in terms of communication satisfaction and communication competence. Communication satisfaction was assessed using four conceptually relevant items selected from Hecht Interpersonal Communication Satisfaction Inventory [35]. The item “I felt I could talk about topic with the other person” was modified from “anything” to “topic” to fit the structured dialogue context, while the remaining three items—“I am generally satisfied with the conversations,” “I enjoy the conversations,” and “These conversations flow smoothly”—were directly adopted. All items were rated on a 6-point Likert scale ranging from “1=extremely disagree” to “6=extremely agree.” Communication competence was evaluated using 6 selected items from the Interpersonal Communication Competence Scale (ICCS) [36], reflecting perspective-taking, emotional awareness, conversational flow, topic management, and sensitivity to nonverbal cues: (1) I can put myself in others’ shoes, (2) I do not know exactly what others are feeling, (3) other people think that I understand them, (4) my conversations are characterized by smooth shifts from one topic to the next, (5) I take charge of conversations I am in by negotiating what topics we talk about, and (6) in conversations, I perceive not only what others say but also what they do not say. Responses were rated on a 4-point Likert scale ranging from “1=extremely disagree” to “4=extremely agree.”

### Secondary Outcome Measures

#### Health-Related Capacity

Health-related capacity was evaluated using measures of health literacy and enablement. Health literacy was assessed with three items from the European Health Literacy Survey Questionnaire (HLS-EU-Q47) [37], including (1) finding information on treatments of illness that concern you, (2) finding information on how to manage mental health problems such as stress or depression, and (3) judging which everyday behavior (such as drinking and eating habits and exercise) is related to your health. Responses were rated on a 4-point Likert-scale ranging from “1=very difficult” to “4=very easy.” A Chinese version of the HLS-EU-Q47 was previously validated to assess health literacy in Chinese populations [38]. Self-care competence was examined by adapting 6 items from the Patient Enablement Instrument (PEI) to the intervention context: (1) able to cope with life, (2) able to understand your health condition, (3) able to cope with your health condition, (4) able to keep yourself healthy, (5) confident about your health, and (6) able to help yourself. Responses were rated on a 3-point Likert scale: “much better,” “better,” and “same or less.” The original PEI was previously translated and validated in the Chinese population [39].

#### Game Experience

Game experience was evaluated after the intervention using the following five questions about the board game: (1) fun to play, (2) encourage intergenerational communication, (3) enhancement of health knowledge, (4) satisfaction with the mission card design, and (5) overall satisfaction with the experience. Responses were rated on a 4-point Likert scale ranging from “extremely disagree” to “extremely agree.” Participants were also asked about their perceived game engagement on a 10-point scale ranging from very disengaged (0) to very engaged (10).

Demographic data were collected before the intervention, including age, gender, living arrangement, education attained, marital status, religion, and self-reported noncommunicable diseases.

All participants were invited to focus group discussions immediately after the game intervention and post survey to collect immediate narrative feedback. The focus groups, consisting primarily of participants from the same age group, were conducted to explore the dialogue experience and views on the board game design. For participants who could not join the focus groups but wished to elaborate on their opinions of the board game, in-depth individual interviews were scheduled within 10 days of their board game experience to minimize recall bias. The focus group discussions and individual interviews lasted approximately 45‐60 minutes, were audiotaped, and participants were invited to freely express relevant ideas on the discussion topics. The qualitative interviews were led by researchers experienced in qualitative data collection using a discussion guide to discuss [40] with three major components, including (1) attitudes toward intergenerational communication, (2) healthy aging, and (3) views on the design and application of the card game.

### Data Analysis

A quantitative analysis of the survey responses was conducted using IBM SPSS (version 29.0). Sample characteristics were reported as descriptive statistics. Items related to intergenerational communication satisfaction, communication competence, health literacy, and the patient enablement index were summed to yield total scores, whereas paired *t* tests were performed to detect significant changes in target outcomes with 95% CIs. Subgroup analyses by age group were further conducted to examine the impact of the card game across different generations. Cohen *d* of the three target outcomes (intergenerational communication satisfaction, communication competence, and health literacy) was computed using the mean difference before and after the intervention to evaluate the effect sizes, and Hedges correction was applied for subgroups with n<20 [41]. Effect sizes were considered small, medium, and large with cutoffs of 0.2, 0.5, and 0.8, respectively [41,42]. Findings related to key game experience components, including “fun to play,” “encourage intergenerational communication,” “enhance health knowledge,” and “satisfied with board game question and mission design,” were first presented descriptively. To further examine the associations between the key game experience components and the likelihood of reporting higher levels of overall satisfaction and game engagement, multivariate binary logistic regression models were conducted. For these analyses, the original Likert-scale or continuous scale outcome variables were dichotomized into “higher” vs “lower” satisfaction or engagement. A higher level of satisfaction was defined as a response of “strongly agree” on the 4-point Likert satisfaction scale, while a higher level of engagement was defined as a score of 9‐10 on the 0‐10 engagement scale. The reported odds ratios (ORs) and corresponding 95% CIs were adjusted for age group in the models. A *P* value of <.05 was considered statistically significant.

Audiotapes recorded during the qualitative sessions were transcribed verbatim, and the transcripts were managed with Microsoft Excel. Experiences related to the game in three aspects, including intergenerational communication, health-related capacity, and overall playing experience, were identified by thematic analysis. The themes and quotes were identified with reference to the discussion guide, and the transcripts were coded twice by 2 independent reviewers to develop the study codebook. With the emergent and recurrent themes identified from the transcripts, these codes were checked and discussed among the independent reviewers to enhance consistency throughout the analysis. Codes were discussed among the research team in case of disagreements. Excerpts were quoted to illustrate the findings uncovered from the qualitative phase.

### Ethical Considerations

The tabletop game intervention is a component of a social isolation project approved by the Chinese University of Hong Kong Survey and Behavioral Research Ethics Committee (SBRE-21‐0870). Before the game intervention, informed consent was obtained from participants for participation in the game, surveys, qualitative interviews, and audiotaping.

## Results

### Demographics

A total of 81 participants were enrolled in the tabletop game, and 68 completed the intervention and evaluations, yielding a response rate of 84%. A total of 19 game groups were arranged, each involving 3‐5 participants with at least 1 participant from each of 3 age groups. Participants were distributed across the 3 age groups (Table 1). The majority of participants were female (82.4%), living with others (61.8%), had a tertiary education level or above (55.4%), were single (47.1%), and had no religious belief (52.9%). About half of the participants reported having a communicable disease (48.5%). After the intervention and post survey, 52 of 68 (76.5%) participants were interviewed in 8 focus groups and 3 individual interviews. Only 1 focus group was mixed-aged, and 3 individual interviews were arranged due to the unavailability to join the focus group. There was no difference in the direction or richness of sharing; therefore, all focus groups and individual interviews were combined for data analysis. Regarding the characteristics of the interviewees across the 3 age groups, a higher proportion with tertiary education, who were single, who had no religious belief, and who had no chronic disease was observed among the youth, while a higher proportion living alone and being widowed or divorced was observed in the middle-aged and older adult groups. The majority of participants were female (80.8%) across all 3 age groups.

**Table 1.** Demographic characteristics of respondents.

Demographic	Total (n=68), n (%)	Young (n=23), n (%)	Middle-aged (n=14), n (%)	Older adults (n=31), n (%)
Age range (years)	19‐92	19‐33	46‐64	66‐92
Sex				
Male	12 (17.6)	7 (30.4)	1 (7.1)	4 (12.9)
Female	56 (82.4)	16 (69.6)	13 (92.9)	27 (87.1)
Live status				
Living alone	26 (38.2)	1 (4.4)	4 (28.6)	21 (67.7)
Living with others	42 (61.8)	22 (95.6)	10 (71.4)	10 (32.3)
Education level				
Primary or below	7 (10.3)	0 (0.0)	0 (0.0)	7 (23.3)
Secondary	23 (33.8)	1 (4.3)	5 (35.7)	17 (56.7)
Tertiary or above	37 (54.4)	22 (95.7)	9 (64.3)	6 (20.0)
Relationship status				
Single	32 (47.1)	19 (82.6)	3 (21.4)	10 (32.3)
Married or stable relationship	18 (26.5)	3 (13.0)	7 (50.0)	8 (25.8)
Widowed or divorced	18 (26.5)	1 (4.3)	4 (28.6)	13 (41.9)
Religion				
No	36 (52.9)	17 (73.9)	3 (21.4)	16 (51.6)
Yes or traditional worship	32 (47.1)	6 (26.1)	11 (78.6)	15 (48.4)
Self-reported noncommunicable disease				
No	35 (51.5)	21 (91.3)	8 (57.1)	6 (19.4)
Yes	33 (48.5)	2 (8.7)	6 (42.9)	25 (81.6)

### Intergenerational Communication

There was a significant improvement in the participants’ self-ratings of communication satisfaction, with a mean difference of −2.76 (95% CI −3.66 to −1.87; Table 2). Participants across all 3 age groups showed significant improvement after the intervention, with mean differences of −2.52 in young adults, −3.93 in middle-aged adults, and −2.42 in older adults. The largest effect size was observed in the middle-aged group (Cohen *d*=1.13). For participants’ ratings of communication competence, significant improvement was also observed after the intervention, with a mean difference of −1.04 (95% CI −1.71 to −0.37). Improvement was shown across all 3 age groups, but statistically significant improvement was observed only among older adults, with a mean difference of −1.32 (95% CI −2.52 to 0.12). Comparing the 2 communication dimensions, a larger effect size was observed for communication satisfaction (medium effect size of 0.75) than in communication competence (small effect size of 0.38) after the intervention.

**Table 2.** Effectiveness of the intervention on intergenerational communication.

Intergenerational communication and measurement	Total (N=68)	Young (n=23)	Middle-aged (n=14)	Older adults (n=31)
Communication				
Baseline, mean (SD)	18.57 (3.31)	18.48 (2.57)	18.14 (3.53)	18.84 (3.74)
Satisfaction				
Intervention, mean (SD)	21.34 (1.87)	21.00 (2.20)	22.07 (1.73)	21.26 (1.61)
Baseline intervention, mean (95% CI)^a^	−2.76 (−3.66 to −1.87)^b^	−2.52 (−4.02; −1.02)^b^	−3.93 (−5.82; −2.04)^b^	−2.42 (−3.91; −0.93)^b^
Cohen *d^a^*	0.745	0.728	1.131	0.595
Communication				
Baseline, mean (SD)	17.62 (2.73)	18.26 (2.40)	18.43 (2.44)	16.77 (2.91)
Competence				
Intervention, mean (SD)	18.66 (2.33)	19.34 (2.21)	18.79 (2.36)	18.10 (2.34)
Baseline intervention, mean (95% CI)^a^	−1.04 (−1.71 to −0.37)^c^	−1.09 (−2.23 to 0.06)	−0.36 (−1.22 to 0.51)	−1.32 (−2.52 to −0.12)^c^
Cohen *d^a^*	0.377	0.411	0.224	0.404

a Cohen *d* was computed with the mean difference by Hedges correction for n<20.

b Paired *t* test.

c *P*<.05

Qualitative interviews supported these quantitative findings, showing that the intervention facilitated easier and more relaxed communication.

A young participant remarked:

Sometimes, when talking to middle-aged and older individuals, you may not always know what to say. Not everyone is skilled in discussing various topics with others. On the contrary, this approach raises topics for conversation on which everyone can share their opinions. It’s not too tense, and it makes the conversation with them much easier.

[BG22 (E)]

A middle-aged participant further added:

… as players often raise different answers, the contrast between them may stimulate even the shy ones to stay engaged with the tasks and responses. This promotes communication, or even builds the trust for any physical assistance with the action tasks when necessary.

[CUBG35 (B)]

An older adult participant also shared:

The gamification provides a welcoming space to share personal matters (anonymously), which helps players to soothe and relax, especially on sensitive topics that might be difficult to bring up with friends, as you may be uncertain if the friend may spread the word unintentionally.

[BG26]

### Health-Related Capacity

Health literacy significantly improved after the intervention (Table 3). Across the 3 age groups, significant improvements in health literacy with a significant result were observed in the middle-aged (mean difference −0.86, 95% CI −1.60 to −0.11) and older adult groups (mean difference −1.19, 95% CI −1.96 to −0.43) but not in the youth group (mean difference −0.13, 95% CI −0.88 to 0.62). Focusing on the patient enablement effect of the tabletop game intervention, PEI scores significantly increased (Table 3). A comparison of results across the 3 age groups showed that older adults’ PEI (mean 6.10, SD 2.18) was significantly higher than that of the young adult group (mean 4.22, SD 2.59). However, no significant difference was observed in the other comparisons. Details of the comparisons are tabulated in Table 2.

**Table 3.** Effectiveness of the intervention on health enhancement.

Health enhancement and measurement	Total (n=68)	Young (n=23), mean (SD)	Middle-aged (n=14), mean (SD)	Older adults (n=31), mean (SD)
Health literacy				
Baseline, mean (SD)	8.59 (2.29)	10.00 (1.38)	8.93 (2.06)	7.39 (2.33)
Intervention, mean (SD)	9.35 (1.74)	10.13 (1.46)	9.79 (1.48)	8.58 (1.77)
Baseline intervention, mean (95% CI)^a^	−0.76 (−1.22 to −0.31)^b^	−0.13 (−0.88 to 0.62)	−0.86 (−1.60 to −0.11)^b^	−1.19 (−1.96 to −0.43)^b^
Cohen *d*^c^	0.409	0.075	0.624	0.572
Patient				
Intervention, mean (SD)	5.41 (2.78)	4.22 (2.59)	5.86 (3.68)	6.10 (2.18)
Enablement				
One-way ANOVA (*P* value)	.04^b^	—^d^	—	—
Index (PEI^e^)				
Post hoc test, group comparison^f^	1=2; 2=3; 3>1	—	—	—

a Paired *t* test.

b *P*<.05.

c Cohen *d* was computed with the mean difference by Hedges correction for n<20.

d Not applicable.

e PEI: Patient Enablement Instrument.

f For the post hoc test (Bonferroni): 1=young, 2=middle aged, and 3=older adults.

Qualitative data corroborated these results, and a young participant indicated the benefits of the intervention in enhancing health literacy, particularly regarding mental health:

There were several cards that addressed topics like how to cope when you're under a lot of stress, or what might make you feel down emotionally. When discussing these topics, everyone can reflect more on their own experiences. It makes us think about times when we ourselves may have felt a lot of emotional pressure or faced emotional issues. Some of the cards also ask how you would cope with such situations, and everyone can share their experiences. This allows for the exchange of skills and strategies for dealing with various topics.

[BG22 (E)]

A middle-aged participant highlighted:

The youth and I (middle-aged) had no idea of the welfare offered by the healthcare voucher or the senior citizen card; we had to check online immediately to learn the benefits… it is like acquiring new knowledge. It is not that we don’t care, but what we knew was limited, and only then did we realise there are things experienced by the older adults that we lack understanding.

[BG20 (5)]

An older adult participant shared the impact of health literacy:

… when you live to a hundred, you would need the help from a carer (social support), and you can’t take good care of yourself. This feels powerless, and the other player agrees, sharing the same concern (about Health-related Quality of Life in ageing). We are worried that we become incapable of self-care, and this is not the ideal way (of community ageing).

[BG26]

In terms of patient enablement, a young participant noted:

The discussion on the HA Go (mobile application of the Hospital Authority) is important, as it provides information for carers like me, who monitored the health conditions of my family with the HA Go… it is still a means for us to manage health regardless of physical distance...

[CUBG13 (M)]

A middle-aged participant also mentioned:

The tabletop game may empower the participants to cope with their future, such as guiding them to think and plan ahead… the positive planning should be for everyone, and everyone should foster a positive mindset.

[BG18 (4)]

An older adult participant expressed:

Expressing my thoughts with the game play made me feel more at ease… and by drawing the tasks randomly, we may not dodge from the problem, but to face it directly. It feels like a therapy session, and it’s therapeutic.

[BG15 (1)]

### Game Experience

A strong positive experience with the tabletop game was revealed, with responses heavily inclined toward “agree” and “extremely agree” in all 4 aspects of game experience. The highest proportion of participants found that the tabletop game could encourage intergenerational communication, with 51.5% strongly agreeing, followed by “fun to play” (44.1%), “satisfied with mission card design” (30.9%), and “enhanced our health knowledge” (26.5%). The 4 aspects of game experience are reported in Table 4. The association between game experience and engagement, and overall satisfaction is reported in Table 5. It was found that “encourage intergenerational communication” (OR 4.49, 95% CI 1.09 to 18.58) was significantly associated with participants’ engagement in the tabletop game, whereas “encourage intergenerational communication” (OR 6.22, 95% CI 1.03 to 37.37) and “satisfied with mission card design” (OR 10.20, 95% CI 1.46 to 71.37) were significantly associated with the overall satisfaction of the tabletop game.

**Table 4.** Experience of the gamification-based intervention.

Gamification-based intervention component	Higher level of satisfaction, n (%)	Higher level of satisfaction^a^, n (%)	*P* value^b^	Higher level of engagement^c^, n (%)	*P* value^b^
Fun to play					
Agree and disagree	38 (55.9)	5 (13.2)	<.001	18 (47.4)	.001
Strongly agree	30 (44.1)	23 (46.7)	<.001	26 (86.7)	.001
Encourage intergenerational communication					
Agree and disagree	33 (48.5)	4 (12.1)	<.001	14 (42.4)	<.001
Strongly agree	35 (51.5)	24 (68.6)	<.001	30 (85.7)	<.001
Enhance health knowledge					
Agree and disagree	50 (73.5)	13 (26)	<.001	28 (56)	.020
Strongly agree	18 (26.5)	15 (83.3)	<.001	16 (88.9)	.026
Satisfaction with board game questions and mission design					
Agree and disagree	47 (69.1)	9 (19.2)	<.001	26 (55.3)	.026
Strongly agree	21 (30.9)	19 (90.5)	<.001	18 (64.7)	<.05

a A higher level of satisfaction is defined as “strongly agree” satisfaction.

b Chi-square and Fisher exact test were conducted.

c A higher level of engagement is defined as scoring 9‐10 on the engagement question.

**Table 5.** Relationship between gamification-based intervention components and board game experience.

Board game experience	Higher level of satisfaction^a^, adjusted OR^b^ (95% CI)	Higher level of game engagement^c^, adjusted OR^b^ (95% CI)
Intercept	0.07 (0.012‐0.39)^d^	0.57 (0.19-1.75)
Fun to play		
Agree and disagree	Ref^e^	Ref
Strongly agree	4.63 (0.71‐30.02)	2.73 (0.48-15.56)
Encourage intergenerational communication		
Agree and disagree	Ref	Ref
Strongly agree	6.22 (1.03‐37.37)^d^	4.49 (1.09-18.58)^d^
Enhance health knowledge		
Agree and disagree	Ref	Ref
Strongly agree	3.67 (0.24‐56.60)	1.47 (0.18-12.11)
Satisfied with mission card design		
Agree and disagree	Ref	Ref
Strongly agree	10.20 (1.46‐71.37)^d^	1.07 (0.16-7.12)

a A higher level of satisfaction is defined as “strongly agree” satisfaction.

b Adjusted by 3 age groups: 18‐39 years, 40‐64 years, and >65 years.

c A higher level of engagement is defined as scoring 9‐10 on the engagement question.

d *P*<.05.

e Ref: reference group.

Regarding the qualitative findings, participants emphasized the opportunities this game offered for intergenerational interaction. For example, one young participant mentioned:

The tabletop game provides a chance, or a platform, for each generation to interact and communicate. You know how busy the locals are, not to mention having meals together, but it was rare for different generations just to sit together. The card game would offer motivation for different generations to come together.

[BG27]

An older adult participant further added:

The card game encourages activities between the youth, middle-aged, and older adults. With these opportunities, we may interact and get to know each other in a friendly setting. It is nice that we can engage with real friends.

[CUBG30]

In addition, a young participant expressed enjoyment of the game:

I find the game enjoyable. The tasks are simple and easy to complete. As other students shared, there are tasks that are relatively exciting and playful, especially those with a time limit or an action-based format. These game play strategies are challenging yet fun...

[BG25 (C)]

A middle-aged participant also commented on the game’s enjoyable nature:

… the older adults seem to enjoy the action tasks, and it’s such a pleasant scene when everyone engages with the same actions on an intergenerational level.

[CUBG34 (H)]

An older adult participant reflected on the intergenerational engagement:

I think the card game experience is nice, and it’s my first attendance at such a game. The intergenerational engagement seems to be drawing everyone closer, and I feel involved as it includes topics related to my childhood.

[CUBG29]

The game’s design was recognized as a key factor in its success. A young participant mentioned:

I would say, first of all, what impressed me the most was that the rules of the game were very simple… I think the key is to make sure they understand the rules of the game so they are willing to engage. If the rules were too complicated, they would likely spend too much time figuring out what to do and what each element meant. So, I believe ensuring a smooth game operation is crucial for the success of the game experience.

[BG24 (D)]

A middle-aged participant also expressed satisfaction with the game design:

It’s obvious that the card game encourages expansion of social network by providing common topics and activities… By involving adjacent players, this is a great design as there will be no dominating players, but they will share the experience together.

[BG20]

An older adult participant also acknowledged the game design:

Introducing oneself through playing cards makes it more interesting for everyone listening. By using this relaxed atmosphere, it allows everyone to get to know each other better.

[CUBG25]

## Discussion

### Principal Findings

This intergenerational intervention yielded notable benefits in intergenerational communication, health literacy, and patient empowerment, especially for older adults. While the majority of gamification interventions for older adults have focused on evaluating the impact on health outcomes [43], the current evaluation provides a comprehensive review of communication and health-related outcomes. In addition, this study highlighted the importance of leveraging gamification to enhance intergenerational communication and ultimately combat social isolation among older adults.

Across all age groups, participants reported significantly higher communication satisfaction post intervention, indicating that the game fostered enjoyable and fulfilling dialogue. While short-term increases in communication satisfaction following an engaging activity are, to some extent, expected and may partly reflect novelty or positive study participation effects, communication satisfaction reflects perceived interaction quality rather than transient mood alone. In this intervention, satisfaction was fostered through structured dialogue, guided turn-taking, and role rotation, which targeted communication processes that can plausibly generalize beyond the immediate gameplay context. Nevertheless, sustained effects cannot be inferred from the present design and require confirmation through longitudinal follow-up. Notably, only older adults showed a significant improvement in their communication competence scores, whereas younger and middle-aged adults did not. One explanation lies in baseline differences: older participants may have had fewer opportunities for rich social interaction before the study due to the prevalence of social isolation among Hong Kong’s older population. Indeed, as of 2021, nearly 190,000 adults aged 65 years and older were living alone in Hong Kong, reflecting a 60% increase since 2011 [44]. Such isolation is linked to declines in communication skills and cognitive function [45]. By facilitating regular, meaningful conversations, the game likely helped older participants practice and regain communication competencies. This aligns with evidence that increasing social interaction can improve older adults’ performance on verbal communication tasks [46]. In contrast, younger adults—who typically have more daily social communication—may have been closer to a ceiling in communication ability, limiting observable gains.

Differential effects on health literacy were also observed. Middle-aged and older participants showed significant improvements in health literacy after gameplay, whereas the youth group did not. Younger adults likely entered the study with higher baseline health literacy, given their greater exposure to education and digital information, and thus had less room for measurable improvement. Older adults, on the other hand, often start with lower health literacy and greater health knowledge gaps. The game’s health literacy module provided an engaging, accessible means of learning health information, resulting in tangible knowledge gains for these groups. This finding is consistent with prior research showing that game-based interventions can substantially increase health-related knowledge [47]. Moreover, intergenerational learning theory suggests that structured dialogue between generations creates opportunities for mutual education and attitude change [48]. During the health literacy tasks, younger players might have shared digital health information or taught health concepts, benefiting their older counterparts. Reciprocally, older adults may have shared personal health experiences, which could enrich understanding for young and middle-aged adults. The middle-aged group, interestingly, also improved in health literacy, suggesting that those in midlife, often caring for both children and older adult parents, found the health knowledge and cross-generational communication practice directly relevant and empowering for their caregiving and self-care roles.

Importantly, the older group showed a significantly higher PEI score post intervention than the youth group, with no significant differences between the other groups. The high PEI score in older adults suggests that the game may have enhanced their confidence and sense of “enablement” in dealing with health issues. Several mechanisms could explain this. First, older participants likely felt a sense of accomplishment and agency by contributing actively to group tasks, such as sharing life experiences or completing challenges. Being listened to by younger peers may have bolstered their self-esteem and sense of purpose, which, according to Socioemotional Selectivity Theory, is highly valued in late life [49]. The theory posits that as people age and perceive a shorter future time horizon, they prioritize meaningful social interactions and emotional fulfillment. Importantly, this does not imply that brief interactions with unfamiliar partners function as strong-tie relationships; rather, the intervention may have uncovered interaction contexts characterized by emotional affirmation, mutual respect, and psychological safety, which align with the socioemotional qualities prioritized in later life. In our study, the older adults’ disproportionate gains in communication ability and health literacy could be a good start toward confidence-building in intergenerational interaction and a strong sense of enablement. Moreover, the opportunity to impart wisdom and bond with younger people likely fulfilled older adults’ socioemotional goals, thereby maximizing their engagement and leading to greater gains from the program. In contrast, younger participants generally have a more future-oriented outlook and myriad social connections; while they enjoyed the game, as evidenced by improved satisfaction, they may not have experienced the same degree of program gains or personal empowerment.

The outcome was a unanimous perception across all age groups that the game strengthened intergenerational communication and was well-designed, fun, and educational. These positive subjective evaluations are noteworthy given the challenge of designing content appealing to diverse ages. By using gamification—points, challenges, and playful interactions—the intervention kept participants intrinsically motivated. Gamification theory holds that introducing game elements into nongame contexts (such as health education) can enhance user engagement and motivation [50]. Consistently, our findings show high engagement and satisfaction scores, indicating that the game format was indeed engaging. Prior studies in older adults have also found that appropriately designed card games can acutely boost mood and well-being during play [51]. Our intergenerational game seems to have capitalized on these effects, creating an enjoyable learning environment that felt less like a health workshop and more like a social gathering.

The association analyses provide further insight into what drove the intervention’s success. We found that participants who felt the game “encouraged intergenerational communication” were significantly more likely to report higher engagement in the activities and greater overall satisfaction, reinforcing the importance of perceived interaction quality as a proximal outcome. In other words, when players perceived that the core purpose of the game, bridging generations, was being achieved, their involvement and satisfaction were maximized. This underscores the importance of making the intergenerational benefits salient—participants who recognized the value in connecting with other ages likely invested more effort and attention, potentially creating a virtuous cycle of engagement and positive experience. We also found that satisfaction with the game design was strongly correlated with overall satisfaction. This highlights the role of user-centered design, encompassing elements such as clear rules, culturally relevant content, and balanced difficulty across modules, which likely enhanced enjoyment. From a gamification perspective, well-designed game mechanics (eg, rotating tasks, creative challenges, and immediate feedback through group discussion) can fulfill psychological needs such as autonomy, competence, and relatedness, leading to higher satisfaction [52].

### Limitations

First, although the inclusion criteria were broad, participants voluntarily enrolled in the intervention, and the majority were female, which may not be representative of all eligible individuals; selection bias may therefore limit generalizability. However, the qualitative interviews provided in-depth insights that may help inform the design of a larger-scale randomized controlled trial. Second, intergenerational communication outcomes were assessed using selected items from validated instruments rather than the complete scales. While this may limit psychometric completeness, the selected items were purposefully chosen to reflect communicative processes relevant to brief, structured intergenerational interactions within a gamified setting. Accordingly, findings should be interpreted with appropriate caution. Full-length scales include items less applicable to this context, and their use may compromise relevance and participant engagement. Third, some outcomes were dichotomized for analysis due to skewed response distributions, which may have reduced variability and statistical power and potentially obscured differences in participants’ responses. Fourth, the sample size for the middle-aged group was comparatively smaller than that of the other 2 groups, although the minimum sample size requirement was met. This imbalance may have limited statistical power for 3-group comparisons. Finally, a 30-minute game duration was recommended, but this was not implemented strictly to sustain positive game dynamics. Variations in actual game time may influence comparisons across groups. However, these variations may also reflect natural engagement patterns in intergenerational communication settings.

In conclusion, the intergenerational card game intervention in Hong Kong demonstrated an effective approach to enhancing communication satisfaction, health literacy, and health enablement, particularly benefiting older adults in an aging, isolated population. By providing an enjoyable and structured platform for cross-generational dialogue, the game enabled meaningful engagement and mutual learning between age groups, fostering high levels of participant engagement. These findings underscore the potential of playful, socially embedded interventions to complement traditional health care services by addressing psychosocial determinants of health. Given its low cost, scalability, and high acceptability, this approach holds promise as a public health tool in aging societies worldwide. Future research should further examine the tool’s psychometric properties and extend this work through randomized controlled trials or longitudinal studies to assess longer-term impacts on health behaviors, clinical outcomes, and broader dimensions of social connectedness.

## Abbreviations

HLS-EU-Q47: European Health Literacy Survey Questionnaire
ICCS: Interpersonal Communication Competence Scale
LOUE: Listening, Observing, Understanding, and Expression
OR: odds ratio
PEI: Patient Enablement Instrument
WHO: World Health Organization
